# Effect of an Emollient Emulsion Containing 15.0% of Caprylic/Capric Triglyceride on the Urocanic Acid of the Stratum Corneum

**DOI:** 10.3390/life13040876

**Published:** 2023-03-25

**Authors:** Alicio Vitorino de Souza Neto, Débora Quintas Balla, Thalita Marcilio Candido, Catarina Rosado, André Rolim Baby, Fabiana Vieira Lima Solino Pessoa

**Affiliations:** 1 Department of Health Sciences, Faculty of Pharmacy, Federal University of Espírito Santo, São Mateus 29932-540, Brazil; 2 Department of Pharmacy, Faculty of Pharmaceutical Sciences, University of São Paulo, São Paulo 05508-900, Brazil; 3 CBIOS—Research Center for Biosciences and Health Technologies, Lusófona University Lusófona’s, 1749-024 Lisbon, Portugal

**Keywords:** urocanic acid, TEWL, hydration, stratum corneum, HPLC

## Abstract

Natural moisturizing factor (NMF) includes several compounds in the stratum corneum (SC), among them, urocanic acid (UCA). Ultraviolet (UV) exposure turns the *trans*-UCA of the SC into its *cis* isomer. We investigated the impact of a topical emollient emulsion treatment on the UCA isomers of the SC exposed to artificial UV stress. Aliquots of emollient emulsion were applied in healthy subjects for 2 h on delimited areas of the volar forearm, then, the SC was removed by tape stripping. Tapes were irradiated in a solar simulator chamber and a high performance liquid chromatograph was used to quantify UCA isomers from stripped SC extract. The amount of both UCA isomers were almost twice higher in the SC treated with the emollient emulsion. We also observed that the UV irradiation elevated the amount of the *cis*/*trans* UCA ratio on the SC (non-treated and treated), suggesting that the emollient sample was not able to avoid the UCA isomerization. The in vivo tests corroborated with the UCA data obtained ex vivo, since we found an increase in the superficial skin hydration with respective reduction of the TEWL, probably occurring by the occlusion performed by the emollient emulsion containing 15.0% *w*/*w* of caprylic/capric triglyceride.

## 1. Introduction

The natural moisturizing factor (NMF), located in the stratum corneum (SC), is an endogenous mixture of compounds that maintains the homeostasis of the skin moisture. The NMF components include several acids, among them, urocanic acid (UCA). To help the NMF to maintain the skin’s physiological humidity, moisturizers can be used by topical application [1,2].

UCA is an imidazole derivative present in the SC which has different roles besides contributing to the skin’s superficial hydration, being also important to SC pH maintenance [3,4]. The ultraviolet (UV) exposition turns the *trans*-UCA into the *cis*-UCA, which has immunogenic effects. These immunogenic effects include the stimulation of keratinocytes to produce tumor necrosis factor (TNF), and of mast cells to produce histamine and prostaglandin E2 [5].

Emollients are pharmaceutical/cosmetic ingredients able to maintain skin humidity [6,7]. Typically, they can be incorporated into emulsions that could fill the extracellular medium of the outermost layer of the skin with water through bonds, which will work as a protective barrier, being also able to prevent superficial inflammation [8,9]. Some emollients can be found naturally in palm and coconut oils, and one of them is caprylic/capric triglyceride, which is widely used in cosmetics [10]. In this research, we investigated the impact of an emollient emulsion containing 15.0% *w*/*w* of caprylic/capric triglyceride over the UCA isomers present into the SC of subjects exposed or not to artificial UV stress, being the samples obtained by an ex vivo assay (tape stripping). Additionally, we confronted the ex vivo assay with skin attributes determined by Tewameter and Corneometer.

## 2. Materials and Methods

### 2.1. Reagents and Chemicals

*Trans* urocanic acid and the triethylammonium phosphate (TEAP) were purchased from Sigma-Aldrich (St. Louis, MO, USA). Chloridric acid and phosphoric acid were purchased from Dinâmica (São Paulo, Brazil). Acetonitrile (ACN) was purchased from Honeywell (Charlotte, NC, USA,). Cetearyl alcohol (and) dicetyl phosphate (and) ceteth-10 phosphate (Crodafos CES) was donated from Croda (Barcelona, Spain). Caprylic/capric triglyceride and phenoxyethanol (and) caprylyl glycol (Optiphen) were donated from Ashland (São Paulo, Brazil). Nylon syringe filter 13 mm × 0.22 μm were purchased from Sigma Aldrich (São Paulo, Brazil). Reagents were of HPLC or of analytical grade, used as received, and the purified water was produced by Merck Millipore Mili-Q Simplicity UV.

### 2.2. Emollient Emulsion

The emulsion containing the emollient and applied to the subjects’ forearms is described in Table 1.

### 2.3. Legal and Ethical Aspects of In Vivo Tests

Health subjects were included in the tests. Procedures and possible collateral effects were explained and informed through the Informed Consent Form. The study was performed according to the Helsinki Declaration and it was approved by the relevant ethics committees. Participants were instructed to not apply topical products in the day before the assay in the area to be tested [11].

### 2.4. Chromatographic Conditions

UCA was quantified by high performance liquid chromatography (HPLC) (Shimadzu, Barueri, Brazil) with diode array detector and CTO-20A column oven, according to an adapted method [12]. A column C18 25 cm× 4.6 mm × 5 mm particle size and a pre-column 10 μm × 4.6 mm (Shimadzu, Barueri, Brazil) were used. The detector was set at 268 nm (1). Mobile phase was an isocratic elution of TEAP 0.01 M and ACN (98:2, pH 3), at a flow rate of 1.0 mL/min (25 °C).

#### UCA Quantification

The analytical method parameters, like linearity, detection and quantification limits, precision, accuracy and specificity were established prior to the quantification of UCA isomers [13,14,15].

To determine the method linearity, three independent runs of the *trans*-UCA were performed from a *trans*-UCA standard solution (1.0 mg/mL) and diluted in the following concentrations in chloridric acid 0.01 N at 0.05, 0.1, 0.25, 1.0 and 2.0 μg/mL. The *cis*-isomer was obtained from *trans*-UCA solution irradiated at 9800 kJ/w^2^ in an UV simulator chamber Atlas Suntest CPS+ (Pittsburgh, PA, USA). The detection limit (DL) and the quantification limit (QL) were calculated according to Equations (1) and (2).

DL = (3δ)/S(1)

QL = (10δ)/S(2)

δ = the standard deviation of the response;

S = the slope of the calibration curve.

For the precision and accuracy, nine determinations were used (three concentrations in replicates of three for each sample). The relative standard deviation (RSD) was calculated (Equation (3)). The accuracy was calculated using relative standard error (RSE) (Equation (4)).

RSD = (ST/DMC) × 100(3)

ST = standard deviation;

DMC = determined mean concentration.

RSE = ((DCM − NV)/NV) × 100 (4)

DMC = determined mean concentration;

NV = nominal value.

To obtain the method specificity, runs of the dilutions of virgin tapes and emollient emulsion were compared to UCA isomers’ chromatograms.

### 2.5. SC Extraction (Tape Stripping)

The SC treatment was performed after the subjects were acclimatized in the experimental conditions (15–20 min, 22 ± 1 °C, 40%–60% relative humidity). Aliquots of emollient cream (2.0 mg/cm^2^) were applied to different areas in the forearms, randomly, and kept for 2 h before extraction of the SC by tape-stripping. After that, SC was removed with propylene-made transparent adhesive tapes (Scotch Magic 3M, Sumaré, Brazil) and samples were artificially irradiated. A Suntest^®^ CPS+, Atlas, Linsengericht, Germany, equipped with a xenon lamp (1500 W) and an optical filter simulating solar radiation was used (wavelengths above 290 nm; 2753 KJ/m^2^) to irradiate the samples of the stripped SC. A total of 10 tapes were removed subsequently in the same area. Negative control was SC without treatment [16,17]. Collected tapes from same areas were immersed in 5.0 mL hydrochloric acid 0.001 N and shaken for 60 s by a vortex, followed by 20 min in an ultrasound bath. The samples were filtered in a syringe filter for posterior HPLC quantification of UCA isomers, according to the method described in Section 2.4 [16,18].

### 2.6. Skin Barrier Function

The transepidermal water loss (TEWL) was performed in a Tewameter TM 300 (Courage-Khazaka GmbH, Köln, Germany), according Pinnagoda et al. (1990). After apparatus stabilization, TEWL (g/m^2^/h) of the subjects was measured after 1 min on the non-treated and treated skin areas where the emollient cream was applied for 2 h. Skin moisturization was assessed by a Corneometer CM825 (Courage-Khazaka GmbH, Köln, Germany) using the same TEWL protocol. Measurements were performed in triplicate [19].

### 2.7. Data Analysis

GraphPad software version 9.5 (GraphPad Software, Inc., San Diego, CA, USA) was used to treat the results by Wilcoxon matched-pairs signed rank test or two-tailed Student *t* test, when appropriate, according to normality test (Shapiro–Wilk). Results indicative of significance were considered as *p*-values less than 0.05 (*p* < 0.05).

## 3. Results and Discussion

In the last years, scarce studies have been conducted to quantify the UCA isomers by HPLC, and they were especially dedicated to investigating skin disorders [12,17,18,20,21]. Through the proposed and adapted ex vivo method, it was possible to determine each of the UCA isomers in subject SC samples by a non-invasive method with a robust analytical tool. The chromatographic method showed retention times at 4.0 and 5.5 min for the *trans* and *cis*-UCA, respectively. The linearity was verified through the analytical curve and correlated parameters that are shown in Table 2.

Both isomers, in their concentration intervals, generated acceptable values of coefficient of correlation (r > 0.99) by linear regression analysis. The precision for *trans* and *cis*-UCA was in the range of 5%, being considered satisfactory, as well as the accuracy. The analytical method presented specificity since tapes and emollient emulsion dilutions did not interfere with the separation and quantification of the analyte isomers.

**Figure 1:**
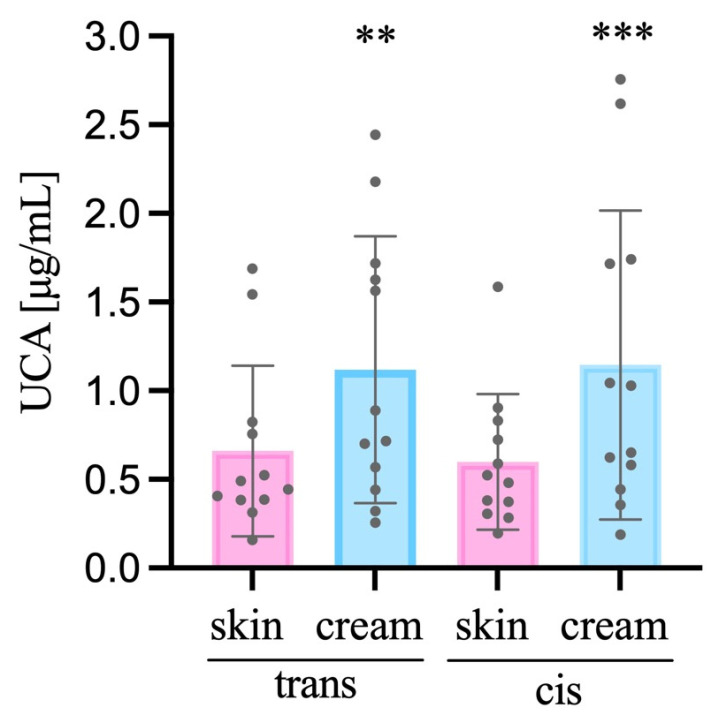
Amount of *trans* and *cis* UCA in SC versus SC + emollient emulsion. ** *p* = 0.00; *** *p* < 0.001, n = 12.

From the sample obtained of tape-stripped non-treated SC, the *trans*-UCA concentration found within the 10 tapes/subjects was 0.6601 ± 0.4817 μg/mL, while in the SC treated with the emollient emulsion treatment was 1.1186 ± 0.7525 μg/mL, being the ratio equal to 1.69 (*trans*-UCA SC treated/non-treated SC). Considering the *cis*-UCA, non-treated SC samples had a concentration of 0.5985 ± 0.3816 μg/mL, and the one found in the treated SC was 1.1452 ± 0.8705 μg/mL (ratio of 1.91; *cis*-UCA SC treated/skin without cream (non-treated SC)). Figure 1 illustrates the *trans*- and *cis*-UCA quantified among the SC samples–Wilcoxin matched-pairs signed rank test (two tailed, n = 12, α = 0.05).

**Figure 2:**
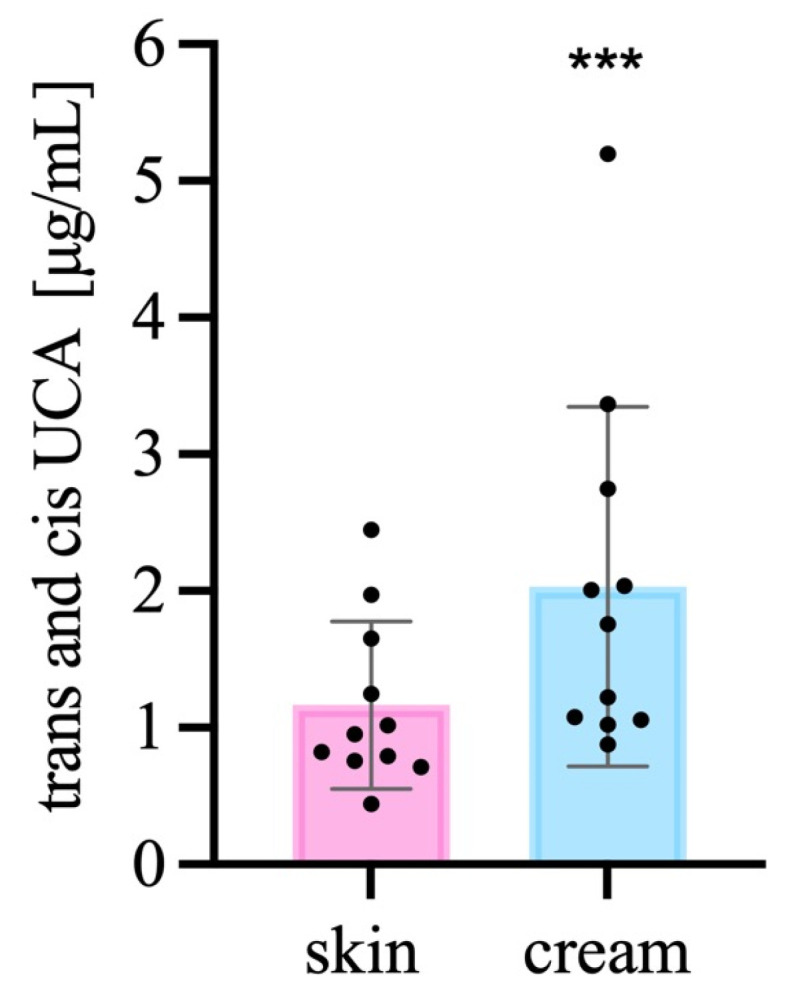
Total UCA quantification in the SC and SC + emollient emulsion. *** *p* < 0.001, n = 12.

Total UCA concentration (the sum of the *trans*- and *cis*-UCA) from the SC samples generated a significant increase when the emollient emulsion was applied to the subjects’ forearms in comparison to the non-treated SC (Wilcoxin matched-pairs signed rank test, two tailed, n = 12, α = 0.05) (Figure 2). Our findings were in agreement with the specialized literature, since some types of emollients are known for the ability to form a lipid film over the skin surface, reducing TEWL, increasing hydration [22,23], and our sample contained 15.0% *w*/*w* of caprylic/capric triglyceride, a considerable concentration, being the emollient skin occludent agent [6,10,24]. The concentrations of both isomers of UCA almost doubled in the SC treated with the emollient emulsion. Our results indicated that the application of the emollient sample, for 2 h, increased the UCA concentration in the outermost layers of the SC; these results agreed with the ones from Teixeira et al. (2014), when the UCA was analyzed as a biomarker for the NMF under 30 days of cosmetic product use, and had an increase of 38.5% [25].

**Figure 3:**
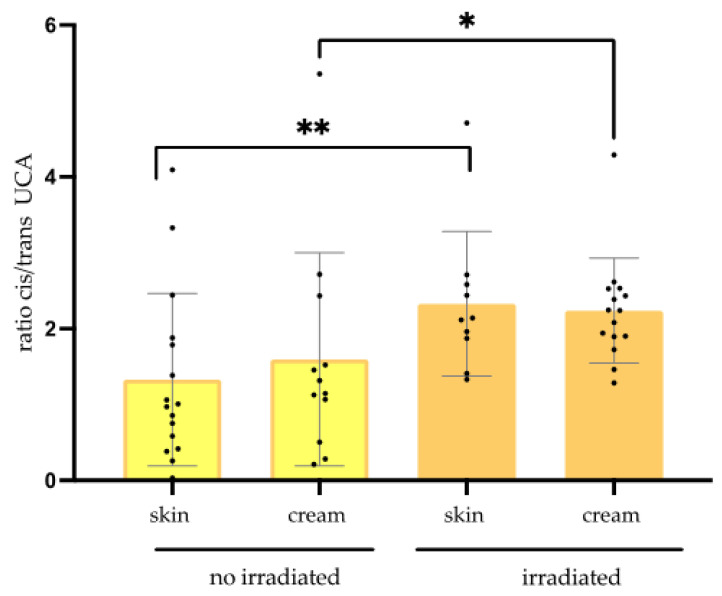
Comparison for *cis*/*trans* UCA ratio in function after UV stress. *p* = 0.266 (no irradiated skin x cream); *p* = 0.910 (irradiated skin x cream); ** *p* = 0.004 (skin before and after UV); * *p* = 0.014 (cream before and after UV); n = 12.

The influence of artificial UV irradiation was also evaluated over the SC obtained by the tape stripping method. Regarding the *cis*/*trans* UCA ratio, before and after the UV stress, in Figure 3, we could observe that UV irradiation elevated the mentioned ratio on the SC (non-treated and treated), suggesting that the emollient sample was not able to avoid the generation of *cis*-UCA, i.e., the isomerization was not controlled. In contrast, the same sample after UV exposure presented an increase of the *cis*/*trans* UCA ratio (SC and SC irradiated; cream and cream irradiated).

**Figure 4:**
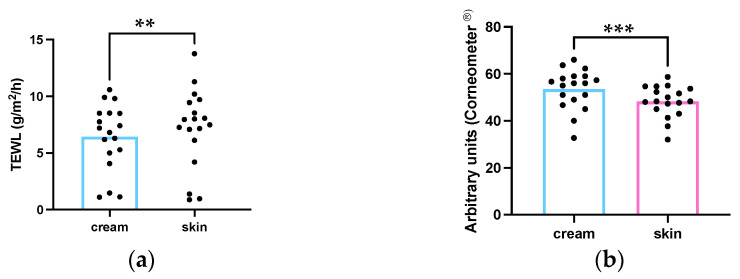
Influence of the emollient emulsion in the skin barrier function: (**a**) TEWL (**b**) skin superficial hydration. ** *p* = 0.005 (**a**); *** *p* < 0.001 (**b**); n = 18.

The emollient emulsion was able to in vivo increase the superficial skin hydration and to decrease the TEWL. Results were analyzed comparing the basal values from each site and after 2 h contact with the emollient emulsion (Figure 4). The skin barrier is a function influenced by the SC architecture, production and quality of the NMF and the level of corneocyte maturation [6,24]. Moisturizers are the common name used for cosmetics that attend to basic needs of consumers and they usually improve skin smoothness, softness, superficial hydration and appearance. Emollients form a film that total or partially occludes the surface of the cutaneous tissue and improves the rate of barrier repair by decreasing TEWL [1,2]. Caprylic/capric triglyceride is classified as an occlusive ingredient that can improve skin moisturize [6,24]. The emollient emulsion contained 15.0% *w*/*w* of caprylic/capric triglyceride which can be attributed the properties of improving the skin barrier function. 

## 4. Conclusions

The HPLC method was successfully used to quantify the UCA isomers from subjects’ SC obtained by tape stripping, a non-invasive method. The amounts of both UCA isomers were almost twice as high in the SC treated with the emollient emulsion. We observed that the UV irradiation elevated the amount of the *cis*/*trans* UCA ratio on the SC (non-treated and treated), suggesting that the emollient sample was not able to avoid the UCA isomerization. The in vivo tests corroborated with the UCA data, since we found an increase in the superficial skin hydration with respective reduction of the TEWL, probably occurred by the occlusion performed by the emollient emulsion containing 15.0% *w*/*w* of caprylic/capric triglyceride.

## Figures and Tables

**Table 1:** Quantitative and qualitative (% *w*/*w*) composition of the emulsion.

Ingredients	Composition % *w*/*w*
Cetearyl alcohol (and) dicetyl phosphate (and) ceteth-10 phosphate (Crodafos CES)	6.0
Caprylic/capric triglyceride	15.0
Phenoxyethanol (and) caprylyl glycol (Optiphen)	1.0
Purified water	78.0

**Table 2:** HPLC analytical parameters for UCA quantification.

Parameters	Isomer	Results
Concentration range (μg/mL)	*trans*	0.050–2.000
	*cis*	0.035–0.708
Slope	*trans*	116,218
	*cis*	67,751
Intercept	*trans*	1054.2
	*cis*	1289.2
Coefficient of correlation (r)	*trans*	0.9999
	*cis*	0.9997
DL (μg/mL)	*trans*	0.01896
	*cis*	0.01806
QL (μg/mL)	*trans*	0.0632
	*cis*	0.06022

## Data Availability

Not applicable.
